# Expanding the Limits of Computer-Assisted Sperm Analysis through the Development of Open Software

**DOI:** 10.3390/biology9080207

**Published:** 2020-08-05

**Authors:** Jesús Yániz, Carlos Alquézar-Baeta, Jorge Yagüe-Martínez, Jesús Alastruey-Benedé, Inmaculada Palacín, Sergii Boryshpolets, Vitaliy Kholodnyy, Hermes Gadêlha, Rosaura Pérez-Pe

**Affiliations:** 1 BIOFITER Research Group, Higher Polytechnic School of Huesca, Institute of Environmental Sciences of Aragón (IUCA), University of Zaragoza, Ctra. Cuarte s/n, 22071 Huesca, Spain; jyaniz@unizar.es (J.Y.); ipalacin@unizar.es (I.P.); 2 Department of Mathematics, Institute of Mathematics and Applications (IUMA), University of Zaragoza, Pedro Cerbuna 12, 50009 Zaragoza, Spain; alquezar@unizar.es; 3 Department of Computer Science and Systems Engineering (DIIS), Aragón Institute for Engineering Research (I3A), Universidad de Zaragoza, María de Luna 3, 50018 Zaragoza, Spain; 698976@unizar.es (J.Y.-M.); jalastru@unizar.es (J.A.-B.); 4 Laboratory of Reproductive Physiology, Faculty of Fisheries and Protection of Waters, South Bohemian Research Center of Aquaculture and Biodiversity of Hydrocenoses, Research Institute of Fish Culture and Hydrobiology, University of South Bohemia in České Budějovice, Zátiší 728/II, 389 25 Vodňany, Czech Republic; sboryshpolets@frov.jcu.cz (S.B.); vkholodnyy@frov.jcu.cz (V.K.); hermes.gadelha@bristol.ac.uk (H.G.); 5 Department of Engineering Mathematics, University of Bristol, 75 Woodland Rd, Bristol BS8 1UB, UK; 6 BIOFITER Research Group, Department of Biochemistry and Molecular and Cell Biology, Faculty of Veterinary Sciences, Institute of Environmental Sciences of Aragón (IUCA), University of Zaragoza, Miguel Servet 177, 50013 Zaragoza, Spain

**Keywords:** sperm quality, computer-aided sperm analysis, open-source software, sperm concentration, sperm function, sperm chemotaxis

## Abstract

Computer assisted sperm analysis (CASA) systems can reduce errors occurring in manual analysis. However, commercial CASA systems are frequently not applicable at the forefront of challenging research endeavors. The development of open source software may offer important solutions for researchers working in related areas. Here, we present an example of this, with the development of three new modules for the OpenCASA software (hosted at Github). The first is the *Chemotactic Sperm Accumulation Module*, a powerful tool for studying sperm chemotactic behavior, analyzing the sperm accumulation in the direct vicinity of the stimuli. This module was validated by comparing fish sperm accumulation, with or without the influence of an attractant. The analysis clearly indicated cell accumulation in the treatment group, while the distribution of sperm was random in the control group. The second is the Sperm Functionality Module, based on the ability to recognize five sperm subpopulations according to their fluorescence patterns associated with the plasma membrane and acrosomal status. The last module is the Sperm Concentration Module, which expands the utilities of OpenCASA. These last two modules were validated, using bull sperm, by comparing them with visual counting by an observer. A high level of correlation was achieved in almost all the data, and a good agreement between both methods was obtained. With these newly developed modules, OpenCASA is consolidated as a powerful free and open-source tool that allows different aspects of sperm quality to be evaluated, with many potential applications for researchers.

## 1. Introduction

The analysis of semen quality is critical in reproductive biology. It is routinely performed in human fertility clinics, animal artificial insemination centers for genetic improvement and cryobanks for humans, domestic animals and endangered species, as well as research centers. Semen analysis usually assesses sperm concentration, motility, morphology, and plasma membrane integrity, among other parameters. Evaluation through direct observation under a microscope by a technician [1,2,3] has been improved thanks to the development of computer assisted sperm analysis (CASA) systems [4,5,6,7,8,9,10] and flow cytometry [11,12,13,14,15].

Despite their usefulness, commercial CASA systems are expensive, inflexible because they do not allow access to algorithms or even raw microscope images acquired, and some studies warn of the high variability of their results [16,17,18,19]. Moreover, these commercial systems are frequently not applicable when new challenges arise in research. For these reasons, in recent years, some authors have proposed free open-source alternatives [20,21,22], including the OpenCASA framework developed by our group [10]. OpenCASA integrates several analysis modules in the same software, and provides a stable and scalable tool for sperm quality evaluation. It allows analysis of motility, morphometry and membrane integrity parameters, and the performance of chemotaxis studies [10]. The project not only makes the code wholly available, but it also allows the scientific community to interact using a mailing list and a forum for knowledge sharing, questions and answers, and feedback/suggestions for new improvements. In this work, we have tackled the limitations of computer-assisted sperm analysis systems, through the development of three new modules in the OpenCASA, with several unprecedented applications of CASA systems.

Recent studies provide experimental support confirming the importance of sperm guidance to the oocyte in the fertilization process. Thus, the study of sperm behavior and its ability to react to external stimuli could be used as an indicator of sperm quality, and could help to predict the fertility of a given seminal sample, assisted by computer automation. One of the most studied mechanisms involved in sperm guidance is chemotaxis, or the directed movement of sperm in response to a chemical gradient [23,24]. Due to the lack of a specific tool in both commercial and open source systems, the first version of OpenCASA included a module for sperm guided movement analysis [10]. This was based on sperm motion within a gradient of the chemotactic agent created in a chamber [7,17]. However, in many external fertilizing species, the existence of a stable chemical gradient is scarcely possible, since the fertilization occurs in an open environment during a very short period of time, especially in freshwater species. Thus, an alternative way of evaluating chemotaxis could be analyzing the sperm accumulation near to the source of a chemoattractant being injected from a micropipette or placed inside microbeads, thus mimicking the influence of the oocyte [25]. With the aim of quantitatively evaluating this phenomenon, we have developed the Chemotactic Sperm Accumulation Module. This tool, apart from generating a heat map to visualize the different degrees of cell accumulation, allows one to estimate the percentage of spermatozoa distributed in concentric areas around the chemoattractant agent.

In this context, assessment of the plasma membrane integrity is also an essential requirement for quality analysis of a seminal sample. An intact sperm plasmalemma is required for metabolic functions, capacitation, zona pellucida binding and the acrosome reaction [26]. Thus, OpenCASA includes a module for evaluation of plasmalemma integrity. Nevertheless, the importance of the acrosomal membrane cannot be ignored, since it is involved in the acrosome reaction, thus critical for successful fertilization [27]. Acrosomal enzymes enable the passage of sperm through the zona pellucida. Normally, acrosomes of capacitated spermatozoa react upon exposure to the cumulus cells, zona pellucida, or other substances associated with oocytes [28]. However, various stressors can cause damage to the acrosomal membrane or can provoke a premature acrosome reaction that would prevent fertilization. In a previous study, we developed a fluorochrome combination that allows the differential labelling of five sperm subpopulations according to their plasma and acrosomal membrane integrities related to the sperm functionality [29]. In the present work, the new Sperm Functionality Module was designed for the automatic recognition and quantification of these sperm subpopulations.

The procedure of counting sperm manually under the microscope is costly and prone to errors, especially if there is a large number of cells in the field of view for concentrated samples. We have developed a module that automatically calculates the sperm concentration with OpenCASA. Although this feature is present in most commercial CASA systems, to the best of our knowledge, this is the first free and open software that allows the assessment of this important sperm quality parameter widely employed in clinics and for research (WHO guidelines).

Implementation of these new modules brings fresh capabilities to OpenCASA v2.0, with a diverse set of powerful tools that can be readily employed by laboratories dedicated to semen analysis.

## 2. Materials and Methods

### 2.1. Software Design

OpenCASA is a plugin of ImageJ [30,31]. The new OpenCASA modules have been implemented in Java language, as are the modules of the first version. We have taken advantage of the OpenCASA modular architecture, which facilitates the development of new features by reutilization of previously implemented code, e.g., functions related to the identification of cells in an image or video. The software is hosted at Github (https://github.com/calquezar/OpenCASA/tree/master/executables/v2.0). This platform allows users not only to download the software, but also to be involved in and contribute to further developments. Additionally, a data set has been uploaded to provide a few examples, in order to test the software.

### 2.2. Experimental Design and Statistical Analyses for Validation of the Modules

#### 2.2.1. Chemotactic Sperm Accumulation Module

Semen from fish species was selected for this validation, since it has been shown that their spermatozoa are able to accumulate around a source of attraction (chemoattractants injected by a microneedle). Sperm samples were obtained from 3 different fish species, with or without hormonal injection during the natural reproduction season, in accordance with fisheries practices. The species were rainbow trout (*Oncorhynchus mykiss*), sterlet (*Acipenser ruthenus*) and carp (*Cyprinus carpio*), which were kept in the ponds of the Faculty of Fisheries and Protection of Waters (FFWP) of the University of South Bohemia in České Budějovice. Spermatozoa from these three species differ in size, activation and duration of motility, patterns of motility and reaction to the environment [18], which results in different accumulation responses in the presence of a chemoattractant. All the experiments were conducted at FFWP and CENAKVA (South Bohemian Research Center of Aquaculture and Biodiversity of Hydrocenoses), following the regulations of the local ethics committee of the University of South Bohemia in České Budějovice, and the legislation on animal welfare and experimentation of the Czech Republic. Before the experiments, sperm samples were stored on ice, for no longer than two hours. Activation of motility was carried out by the dilution of 0.1–0.3 µL of sperm, in an open drop (40 µL) of distilled water or specific activation solution (depending on the species) under a light microscope (UB 200i, PROISER, Paterna, Spain) with negative phase contrast. Sperm motility was recorded using a digital video camera (Ul 3130CP-M, IDS, Obersulm, Germany or ISAS digital camera, PROISER), with resolution 800 × 600 or 768 × 576 and 25 frames per second (fps). We used the substances which were shown to have an attractant effect on the spermatozoa of each fish species: ovarian fluid for trout and carp sperm, and egg conditioned water (medium collected after 15 min of incubation with non-destroyed eggs washed of ovarian fluid) for sterlet sperm (unpublished data). These solutions were introduced into the drop, immediately after sperm activation, using the microinjector (CellTram 167 Vario, Eppendorf, Hamburg, Germany) on a holder (Narishige, Tokyo, Japan) and microneedle (pulled from microcapillaries G100, Narishige). In the case of control samples, the injected solution was the same medium, which was used for the activation of sperm motility.

In this study, only typical examples of recorded sperm motility were presented, together with their following analysis with the module, i.e., no statistical processing of the obtained data was done. This module was validated with a case-control approach, where videos of fish sperm in the presence or absence (control) of a known attracting agent were analyzed in order to find differences (pair of video per species). All the videos are available online—see Supplementary Material 1 (chemotactic sperm accumulation module). The presence of a chemoattractant triggered sperm chemotaxis, leading to subsequent sperm accumulation towards a localized region near the source, i.e., the tip of the microneedle that introduced the attracting agent into the medium. For the control condition (without chemoattractant), the sperm swimming behavior was unbiased near to the source, resulting in no accumulation of spermatozoa. Thus, the validation of this module consisted of demonstrating its ability to detect and measure sperm accumulation, or lack of it, in all the empirical conditions.

#### 2.2.2. Sperm Functionality Module

Cryopreserved-thawed semen samples from 10 Holstein bulls were stained using the ISAS^®^3Fun kit (PROISER), as described in [29]. The labelling mix included three fluorochromes: propidium iodide, Hoechst 33342 and carboxyfluorescein diacetate (CFDA); therefore, plasma membrane and acrosomal integrity could be assessed simultaneously in wet samples. An epifluorescence microscope (DM4500B, Leica; Wetzlar, Germany), equipped with a warming stage, a 20× magnification objective, and a standard blue/green/red filter set (B/G/R, excitation: 420–430, 495–515, 570–620 nm), was used to obtain digital images of the fluorescence-labelled spermatozoa. Images were captured using a Canon EOS 600D Digital Camera (Canon Inc., Tokyo, Japan), controlled with EOS Utility software. Sperm were grouped into five sperm subpopulations: (1) IAIM, with intact acrosome and intact plasma membrane (blue spermatozoa with green acrosome); (2) IADM, with an intact acrosome and damaged plasma membrane (red spermatozoa with green acrosome); (3) DAIM, with damaged acrosome and intact plasma membrane (blue spermatozoa); (4) DADM, with damaged acrosome and plasma membrane (red spermatozoa), and (5) IFI, with increased fluorescence intensity (green sperm head and tail) [29].

All spermatozoa in each image were classified in five subpopulations, both manually (visual estimation by an observer) and by the Sperm Functionality Module of the OpenCASA system. A representative sample of the images used are available online—see Supplementary Material 2 (Sperm Functionality Module). Results from the visual and automated methods were compared using the Spearman correlation test, as the data were not normally distributed (D’Agostino and Pearson). Given that the correlation analyzes the relationship between two variables, and not their scale differences, the Bland–Altman test was carried out to study the agreement between the two different measurement systems.

#### 2.2.3. Sperm Concentration Module

Cryopreserved semen samples from 2 Holstein bulls were also used for the validation of this module. Straws with 0.25 mL of frozen semen were thawed for 1 min at 37 °C in a water bath and diluted 1:1500 in a 0.37% formaldehyde solution in PBS, in order to avoid sperm movement. The diluted samples were loaded into a Neubauer improved haemocytometer (Marienfeld, Germany), and were allowed to settle for 2 min. Moreover, 24 images corresponding to different fields out of the grid were recorded under an Olympus BX40 microscope (Olympus Optical Co., Tokyo, Japan), with a 10× magnification negative phase objective.

All spermatozoa in each image were counted both manually (visual estimation by an observer) and by the Sperm Concentration Module of the OpenCASA system. A representative sample of the images used are available online—see Supplementary Material 3 (Sperm Concentration Module). Results from the visual and automated methods were compared using the Pearson correlation as the data were normally distributed (D’Agostino and Pearson). The Bland–Altman test was carried out to study the agreement between the two different measurements.

## 3. Results

### 3.1. Development and Validation of the Chemotactic Sperm Accumulation Module

#### 3.1.1. Development of the Chemotactic Sperm Accumulation Module

**Figure 1:**
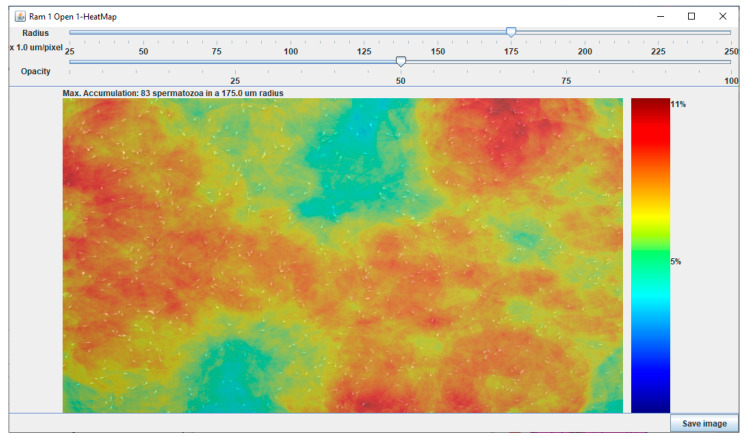
Heat map provided by the Chemotactic Sperm Accumulation Module, showing sperm accumulation. Right bar shows the Jet look up table (LUT). Cold and warm colors represent low and high accumulation values.

This module generates a heat map related to the sperm accumulation values obtained from an image or video of a sperm sample. Taking into account that videos are mainly sequences of images (called frames), the analysis is very similar for both types of files. First, images are preprocessed following these steps: grayscale conversion, histogram equalization and thresholding. Next, ImageJ’s Analyze Particles function is used to detect sperm cells and obtain their location in the image. Once all sperm locations have been stored, spermatozoa in a user-defined distance R are counted for each pixel in the image. The result is a matrix whose dimensions match those of the original image. Since, at the borders of the image, the R-radius circle exceeded the limits of the image, the corresponding sperm counts were adjusted according to the proportion of the circle that remains within the image (see Document S1 for details). The last step consists of making a mapping between the accumulation values and the heat map colors. For this purpose, concentration values (*x*) are linearly scaled between 0 and 255:

(1) $$Scaled\_value\left(x\right)=\left(x-x_{min}\right)\cdot \frac{255}{x_{max}-x_{min}}$$

where *x* is the concentration value to be scaled, and *x_max_*/*x_min_* denotes the maximum/minimum concentration values found in the sample. Then, each scaled value is used as an index to access an ImageJ 256-entry look up table (LUT), called Jet, to return a RGB value. The result is the heat map (Figure 1), where cold and warm colors represent low and high accumulation values, respectively.

In the case of a video, the same procedure described above was applied to each frame of the video, in order to obtain a sequence of heat maps. As this requires more computing power, especially in videos with many frames, two parameters were incorporated to speed up the computation: (I) a sampling factor: a heat map is calculated for every *f* frames, and the intermediate heat map frames are interpolated. In this way, the time required for calculating the heat map in a video is accelerated by a factor f; (II) pixel window: sperm cells are searched and counted only for the center pixel of a W × W window, with W being an odd natural number greater than 1. The expenditure of the process is reduced by a factor of W^2^. As a result of this acceleration, the quality of the resulting video is lower, as windows of W × W pixels are painted with the same value.

In order to monitor the number of spermatozoa that accumulate in a certain area over time, a circular region of interest (ROI) can be drawn, using the mouse while pressing the Shift key. The software shows the real radius of the circle in microns and the number of sperm in this ROI for each frame of the video, both total and relative to the number of spermatozoa in the sample. The same information is also shown for two additional concentric circles, with double and triple radius of the original circle. Thus, changes in the number of sperm in the adjacent areas can be monitored. This information allows the generation of graphs to evaluate the accumulation of the spermatozoa near to the injected attractant in relation to time.

#### 3.1.2. Validation of the Chemotactic Sperm Accumulation Module

**Figure 2:**
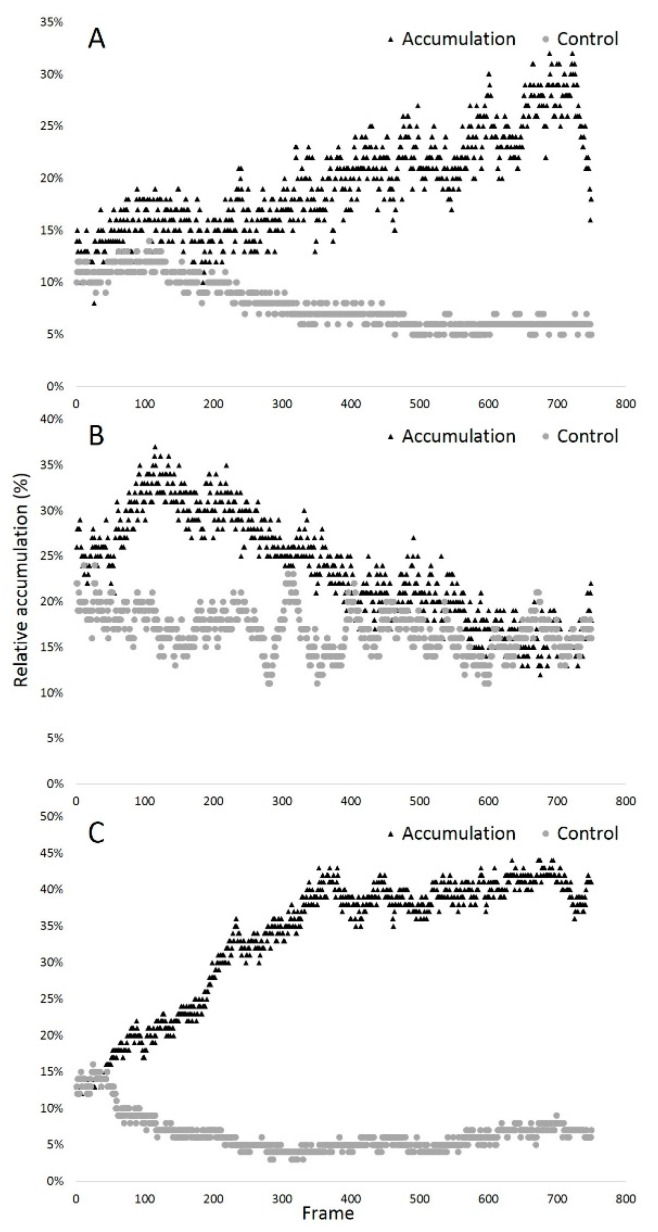
Relative accumulation changes shown by the Chemotactic Sperm Accumulation Module, assessed through individual frame sequences: (**A**) trout spermatozoa; (**B**) carp spermatozoa; (**C**) sterlet spermatozoa.

Six videos corresponding to three case-control pairs were analyzed (Videos S1–S6; coded as 1_1–3_2, where the first number in the name denotes the species (1—trout; 2—carp; 3—sterlet) and the second number is an accumulation (1) or control (2) condition). The resulting heat maps are included in the Videos S7–S12; the name of the file corresponds to the name of the video. In each pair of videos, a ROI with the same radius was used (selected ROI for each of the tested videos are presented in Figures S1–S6; the name of the file corresponds to the name of the video). We have examined, in detail, how the choice of parameters may increase the precision of the results, and we have shown the influence of those parameters in the analysis (see Appendix A). During the observation, the total number of spermatozoa in the vision field may change, so using relative accumulation is the most objective way to demonstrate accumulation dynamics. When videos with an induced chemotactic accumulation effect (case conditions) were recorded (Videos S1–S3), the analysis by the Chemotactic Sperm Accumulation Module clearly indicated cell accumulation. At the same time, analysis of the control videos (Videos S4–S6) did not show any accumulation. These results may be visualized by the graphs presented in Figure 2, where the case-control pair differences are obvious. A good concordance between time series and video heat maps was observed in the first and the third pair of videos. However, in the second pair, we observed a high and very fast accumulation of spermatozoa very close to the tip of the needle, resulting in a significant sperm aggregation. When the cells were aggregated, the software considered the group as one single event, but larger than the size limit established in the module settings, and therefore did not take it into account. Thus, in the graph corresponding to the second pair (Figure 2B), the increasing accumulation of cells suddenly changes to an abrupt decline (around the 90th frame), when the concentration of cells became too high for correct tracking. Accordingly, this limitation should be considered in the analysis.

### 3.2. Development and Validation of the Sperm Functionality Module

#### 3.2.1. Development of the Sperm Functionality Module

Using a specific mix of fluorochromes, the spermatozoa showed different staining patterns according to their acrosome and the plasma membrane integrity. Thus, the spermatozoa were labelled in blue or red, both alone or combined with green (the acrosome). The Sperm Functionality Module analyzes the color of the pixels of each cell. For each pixel, the software uses a table of correspondence, in order to label it in red, green or blue. To do this, a correspondence has been set between each of these colors, and a range of values in the HSV color model, specifically the hue component, H: Red when H > 200 or H < 20, Green when 50 < H < 125, and Blue when 130 < H < 185 (this criteria has been determined empirically). After counting all pixels, for each cell the module computes the ratio of colored pixels as follows:

$$Red_{ratio}=\frac{reds}{total}\;Green_{ratio}=\frac{greens}{total}\;Blue_{ratio}=\frac{blues}{total}$$

**Figure 3:**
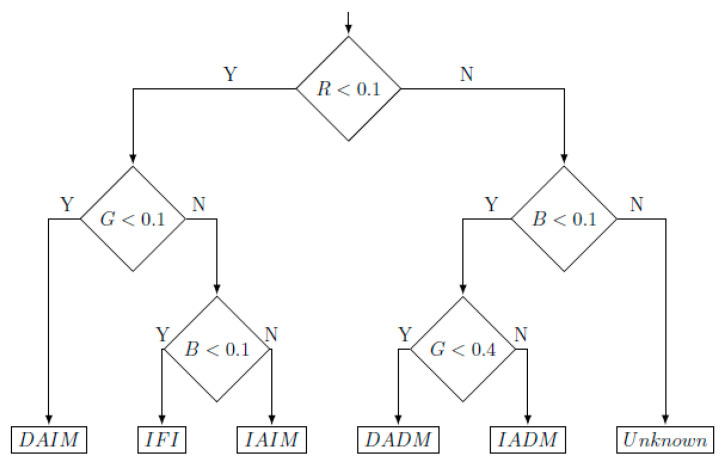
Decision tree used by the Sperm Functionality Module to classify spermatozoa into the different defined cell subtypes, according to plasma and acrosomal membrane integrity.

From these ratios, the program classifies stained spermatozoa into different subtypes, according to the decision tree shown in Figure 3, and labels them with the corresponding nomenclature mentioned in Section 2.2.2.

**Figure 4:**
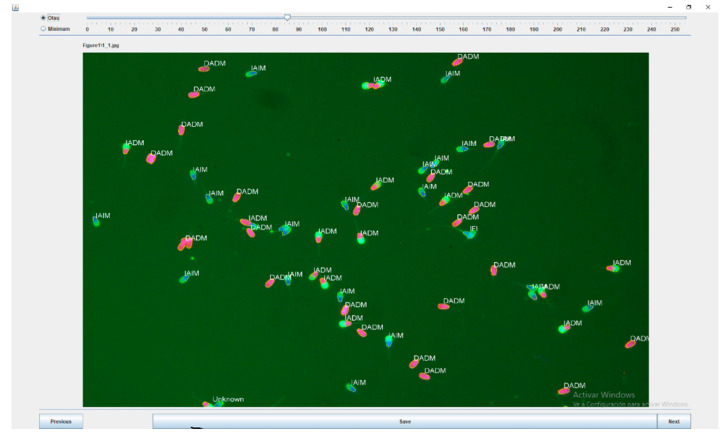
Sperm functionality module output, showing the spermatozoa classification in IAIM (Intact Acrosome and Intact Membrane), IADM (Intact Acrosome and Damaged Membrane), DAIM (Damaged Acrosome and Intact Membrane), DADM (Damaged Acrosome and Damaged Membrane) and IFI (Increased Fluorescence Intensity).

For each image, the module shows the total number of cells, the number and the percentage of each subtype, and the percentages of sperm with acrosomal or plasma membrane integrity (Figure 4).

#### 3.2.2. Validation of the Sperm Functionality Module

In order to validate this module, the percentages of different sperm subtypes according to their staining pattern were evaluated, both manually (visual estimation by an observer) and by OpenCASA. These results were compared using the Spearman correlation and the Bland–Altman test (see Table 1 and Figure S7). The percentages of each sperm subpopulation obtained by both systems were highly correlated (*p* < 0.0001), and showed a very good agreement (bias), except for those spermatozoa with damaged acrosome, but still preserving intact plasma membrane (DAIM). Fortunately, the proportion of spermatozoa of this subpopulation present in the samples is usually low (1.3% in this study), and therefore has little impact on the global results obtained.

### 3.3. Development and Validation of the Sperm Concentration Module

#### 3.3.1. Development of the Sperm Concentration Module

As in the chemotaxis module, the detection of objects in an image requires a preprocessing stage consisting of conversion to grayscale, equalization of histogram, and thresholding. After these steps, ImageJ’s Analyze Particles function is used to detect spermatozoa. In order to correctly identify all cells, it is necessary to specify the thresholding method selected (by default, the software uses the Otsu method), the minimum and maximum sperm size (µm^2^) (depending on the species), and the image scale (µm/pixel). The module computes the concentration of the sperm samples (millions of spermatozoa per mL), based on the dimensions of the counting chamber (width, height, and depth) and the total number of sperm counted. A text box allows users to include the dilution factor in case samples have been diluted before counting, to avoid overlapping.

#### 3.3.2. Validation of the Sperm Concentration Module

Sperm concentration values were obtained manually (visual estimation by an observer) and by OpenCASA. In our experimental conditions, the Otsu thresholding method was selected, since it provided the best results in terms of sperm detection. Results compared using Pearson’s correlation test showed a high correlation (*p* < 0.0001), and a good agreement between both measurement systems was revealed on the basis of the Bland–Altman test (Table 2 and Figure S29).

## 4. Discussion

In this study, three new modules of OpenCASA were developed, aiming to expand the research capabilities of open computerized-assisted sperm quality analysis systems. This work builds on the previous architecture of OpenCASA, and presents a new step towards a truly open source platform for sperm analysis with diverse functionalities. For this purpose, we have taken into account the feedback received by the scientific community since the first version of the software was released [10], which equally demonstrates not only the success of the OpenCASA venture, but also the critical need for an open science approach in the field of reproductive biology [32,33].

A crucial step in the fertilization process is the ability of sperm to move towards the oocyte. Basically, sperm motility is associated with symmetrical (or almost symmetrical) waves produced by flagellum and leading to regular linear motion (or circular motion with high radius). Moreover, many spermatozoa from different species could change the direction of movement in response to any stimuli. This process is commonly associated with temporal local boost of intracellular Ca^2+^ ions in flagellum, leading to formation of highly asymmetrical wave and, as a result, to changes in direction of movement. For mammals and other species, sperm motility usually occurs in a gradient of stimuli, which is often associated with response called hyperactivation or hyperactivation-like motility (for details, see [34]). For this reason, we previously developed a module based on changes in the directionality of the trajectories, in response to a gradient of a chemical substance, temperature, or fluid flow [10], and have introduced the parameter of FD (fractal dimension), which could characterize the sperm trajectory and clearly distinguish the hyperactivation. However, this is restricted to the study of the sperm guidance mechanism in specific species. Sperm from externally fertilizing species, such as freshwater fish, where chemotaxis has been barely studied and demonstrated, show a very short period of motility after activation in water. In the vast majority of freshwater fish species, the motility does not last for more than 0.5–2 min [35]. During such a short time, viscous environments such as the ovarian fluid acting as chemoattractant will not be able to create a spatial gradient. In this case, the most common way to analyze chemotaxis during fertilization in open water is analyzing the sperm accumulation in the vicinity of the stimuli (chemoattractant). Here, we have developed a module that can robustly serve this purpose and evaluate sperm concentration spatially and temporally within an observation field.

The Chemotactic Sperm Accumulation Module is able to recognize and correctly track sperm heads over time, under different conditions (magnification, resolution etc.), to build heat maps of sperm that clearly show sperm accumulation or its absence. Moreover, it provides percentages of sperm accumulating around the stimuli. A comparison between spermatozoa under the influence of a chemoattractant or without it has enabled the validation of this module, based on the quantification of the sperm accumulation. Similar to other CASA systems, it relies on single sperm head detection, and thus has the same image segmentation limitations: when the spermatozoa concentration is very high and sperm collide very often, the module may estimate the accumulation erroneously. In such cases, it is recommended to crop individual frames and analyze them separately as images, using the lowest radius to detect the region where spermatozoa were not tracked. It is worth noting that the module evaluates the “collective” behavior of spermatozoa in the presence/absence of attracting agent, and not the individual reactions of spermatozoa, e.g., trajectory of single cells or changes in flagellar movement, which is an important issue for future development.

Although chemotaxis is a widespread phenomenon in many phyla of animals and plants, sperm attractants have been identified only in some species, generally in marine invertebrates. To the best of our knowledge, this is the first open access tool developed to quantify this phenomenon, and it could be useful in the study of chemotaxis and chemoattractant identification in external fertilization species [36,37]. In addition, the Chemotactic Sperm Accumulation Module could also be applied to study the guided movement of spermatozoa under any other stimulus, such as temperature gradient (thermotaxis [38]), or fluid flow (rheotaxis [39]). Finally, it is worth noting that although OpenCASA software is aimed at the analysis of the seminal quality, some of the modules, such as Sperm Concentration or Chemotactic Sperm Accumulation, are sufficiently general, and can be used for other motile cells, including bacteria, only requiring adjustment of the cell size in the parameter setting screen.

The Sperm Functionality Module allows five sperm subpopulations to be differentiated according to their plasma membrane and acrosomal status, which are essential for sperm function [29]. This technique offers the opportunity to evaluate different attributes simultaneously for individual cells within a sperm population in a short time; therefore, it can greatly improve our current semen quality analysis. To the best of our knowledge, this is the first open computer-assisted system able to automatically classify spermatozoa based on simultaneous multiparameter assessment. Automatic multiparameter sperm classification (e.g., based on plasma membrane, acrosome integrity and/or mitochondrial function) has been predominately based on flow cytometry systems [40,41,42,43]. OpenCASA also has the potential to be adapted to the multiparameter determinations described in these referenced studies. The new Sperm Functionality Module may also be useful for studying the ability of some agents to induce the sperm acrosome reaction [44,45], and to evaluate the effect of various stressors, such as cold-shock or cryopreservation [46,47,48,49], which could provoke a premature acrosome reaction that would prevent fertilization [50,51].

The Sperm Concentration Module has the following advantages when compared to commercial software with the same functionality. First, it is a free software, so that many research groups that do not have access to a commercial CASA system may also determine sperm concentration in a precise and fast way. Second, the software is compatible with different cameras and image file formats, so that no additional equipment is usually required. Third, the same software may be used by different labs, allowing the standardization of the technique. Finally, OpenCASA is more flexible than commercial CASA systems, because it allows access to algorithms, so that adaptations to specific necessities may be undertaken by the different research groups. The results were strongly correlated with visual counting when using a Neubauer chamber, the reference method recommended by the WHO for human sperm evaluation [52]. Nevertheless, this module could also be suitable for other different counting chambers, since it allows users to set the depth of the chamber and the resolution of the image. Thus, the module automatically calculates, from the number of counted sperm, the concentration in millions of cells per milliliter. If the initial concentration of the sperm sample is high and requires dilution to avoid overlapping, the dilution factor can be included after the automated counting is performed in the text box for the sperm concentration of the undiluted sample.

## 5. Conclusions

In conclusion, the three new modules, along with the other four which are already part of the software, make OpenCASA a powerful multifunctional tool, free and open-source, suitable for use in human andrology clinics, assisted reproduction laboratories, animal insemination centers and genetic resource conservation programs.

## Figures and Tables

**Table 1:** Comparison between the percentages of each subtype based on acrosomal and plasma membrane integrity given by manual analysis and by the Sperm Functionality Module included in OpenCASA. Both methods were compared using the Spearman correlation test and the Bland–Altman test.

	Spearman’s Correlation		Bland–Altman
Sperm Subtype	r	*p*-Value	Bias (%)
IAIM	0.801	<0.0001	1.636
IADM	0.827	<0.0001	0.491
DAIM	0.208	n.s.	−0.932
DADM	0.851	<0.0001	−1.551
IFI	0.621	<0.0001	1.854

IAIM: sperm with intact acrosome and intact plasma membrane; IADM, with an intact acrosome and damaged plasma membrane; DAIM, with damaged acrosome and intact plasma membrane; DADM, with damaged acrosome and plasma membrane; IFI, increased fluorescence intensity in the sperm head and tail.

**Table 2:** Comparison between the concentration values given by manual analysis, and by the Sperm Concentration Module included in OpenCASA. Both methods were compared using the Pearson correlation test and the Bland–Altman test.

Pearson Correlation		Bland–Altman
r	*p*-Value	Bias (%)
0.958	<0.0001	−2.407

**Figure A1:**
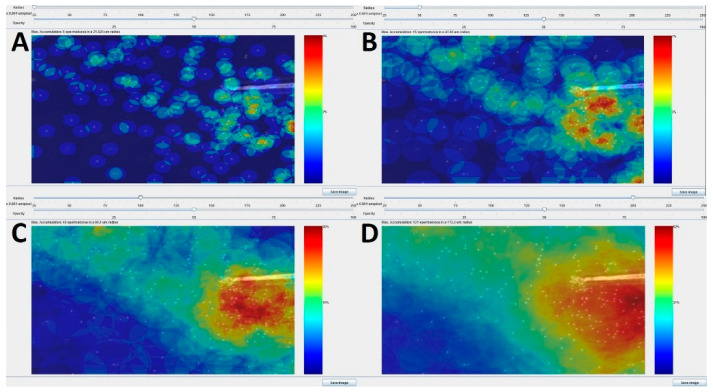
Heat maps provided by the *Chemotactic Sperm Accumulation Module* of the same frame processed with different radius values: (**A**) 25; (**B**) 50; (**C**) 100 and (**D**) 200 pixels. Scale: 0.861 µm/pixel.

**Figure A2:**
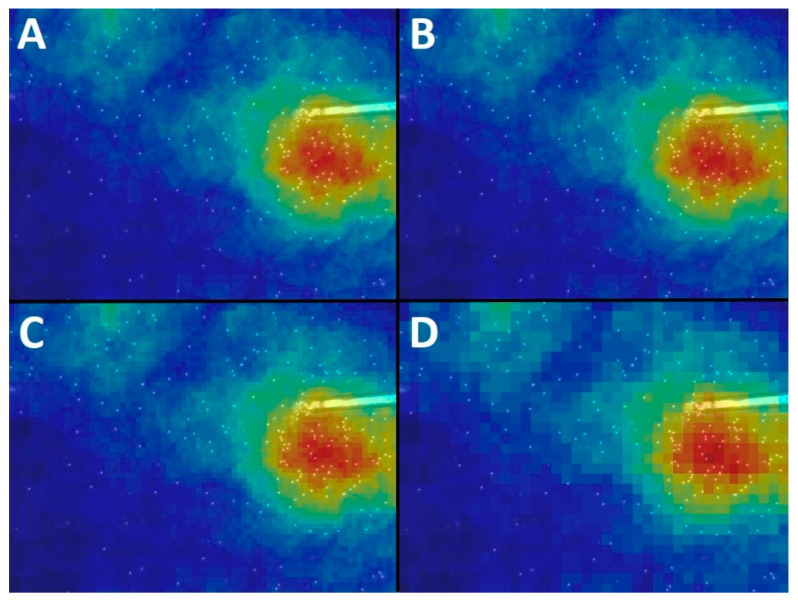
Example of heat map for the same video recorded with different values of the window size: (**A**) 1 pixel; (**B**) 5 pixels; (**C**) 10 pixels; (**D**) 20 pixels. Scale: 0.861 µm/pixel.

## Supplementary Materials

The following are available online at https://zenodo.org/record/3973067#.XyqjvTURVPY. **Suppl. Material 1 *(Chemotactic sperm accumulation module*):** Video S1: Behavior of trout spermatozoa in the presence of an attractant injected by microneedle (1_1).; Video S2: Behavior of carp spermatozoa in the presence of an attractant injected by microneedle (2_1).; Video S3: Behavior of sterlet spermatozoa in the presence of an attractant injected by microneedle (3_1).; Video S4: Behavior of trout spermatozoa in the absence of an attractant. The activation medium is injected by microneedle (1_2).; Video S5: Behavior of carp spermatozoa in the absence of an attractant. The activation medium is injected by microneedle (2_2).; Video S6: Behavior of sterlet spermatozoa in the absence of an attractant. The activation medium is injected by microneedle (3_2).; Video S7: Heat map characterizing trout spermatozoa distribution density in the presence of an attractant injected by microneedle (1_1 HM).; Video S8: Heat map characterizing carp spermatozoa distribution density in the presence of an attractant injected by microneedle (2_1 HM).; Video S9: Heat map characterizing sterlet spermatozoa distribution density in the presence of an attractant injected by microneedle (3_1 HM).; Video S10: Heat map characterizing trout spermatozoa distribution density in the absence of an attractant. The activation medium is injected by microneedle (1_2 HM).; Video S11: Heat map characterizing carp spermatozoa distribution density in the absence of an attractant. The activation medium is injected by microneedle (2_2 HM).; Video S12: Heat map characterizing sterlet spermatozoa distribution density in the absence of an attractant. The activation medium is injected by microneedle (3_2 HM).; Video S13: Example of heat map in the case that 1 frame was used as the interpolating interval during video analysis. (1_1 1f).; Video S14: Example of heat map in the case that 5 frames were used as the interpolating interval during video analysis. (1_1 5f).; Video S15: Example of heat map in the case that 10 frames were used as the interpolating interval during video analysis. (1_1 10f).; Video S16: Example of heat map in the case that 25 frames were used as the interpolating interval during video analysis. (1_1 25f).; Video S17: Example of heat map in the case that a constant maximum of accumulation is used during analysis of the whole video in the presence of attractant. (1_1 constant).; Video S18: Example of heat map in the case that a constant maximum of accumulation is used during analysis of whole video in control conditions (1_2 constant).; Video S19: Example of heat map in the case that a dynamic maximum of accumulation is used for each frame separately in the presence of attractant (1_1 dynamic).; Video S20: Example of heat map in the case that a dynamic maximum of accumulation is used for each frame separately in control conditions (1_2 dynamic); Figure S1: Selected ROI for the Video S1 (1_1); Figure S2: Selected ROI for the Video S1 (2_1). Figure S3: Selected ROI for the Video S1 (3_1); Figure S4: Selected ROI for the Video S1 (1_2); Figure S5: Selected ROI for the Video S1 (2_2); Figure S6: Selected ROI for the Video S1 (3_2); Document S1: Concentration adjustment at the edges of the image. **Suppl. Material 2 (*Sperm Functionality module*):** Figures S7–S15: Examples of the fluorescent images used to validate the *Sperm Functionality module*.; Figure S16: Bland-Altman plots for the percentage of each sperm subpopulation given by manual analysis and by the *Sperm Functionality Module* included in OpenCASA. **Suppl. Material 3 (*Sperm Concentration module*): Figures S16–S28**: Examples of the phase-contrast images used to validate the *Sperm Concentration module*.; Figure S29: Bland-Altman plot for the cell concentration given by manual analysis and by the *Sperm Concentration Module* included in OpenCASA. **Supp. Material 4:** OpenCASAv2.0 plugin.

## Appendix A

### Appendix A.1. Study of the Influence of Parameter Settings on the Results of the Chemotactic Sperm Accumulation Module

The persistent parameters (important settings for the analysis) and calibration should be performed for each specific type of microscope video-recording. This includes camera type, resolution and magnification, among other specifications. They can affect the imaging quality, and therefore the imaging processing output of this module. Calibration of the correct ratio of microns/pixel size is essential as this governs the correct sizing of sperm heads to be detected.

The processing time for the analysis is greatly improved when the appropriate parameter setting for each video-type is employed. It is worth noting that the concentration maps displayed are also affected by the parameter settings as indicated below. Thus, we examine in detail the best choice of parameters to increase the precision of the results and to show the influence of those parameters on the analysis.

### Appendix A.2. Radius

This parameter can be easily changed using the slider at the top of the window (see Section 3.3). Estimation of the optimal radius around the sperm head before analysis is compulsory and can be done in a single frame. Examples of heat maps obtained using different radius are presented in Figure A1. The choice of optimal values is closely associated with the camera resolution and magnification used, while it may still be affected by cell size, sample concentration, etc. In the examples presented, the best choice for the radius was 100 pixels, due to the resulting good visualization, with a clear distribution of colors throughout the heat map.

### Appendix A.3. Window Size

This parameter is crucial for the analysis, affecting not only the resolution of the final heat map, but also the performance of the computation, i.e., the time spent to calculate the results. This parameter specifies the window in which “heat” from each spermatozoon will be calculated. If the window is equal to 1, the module calculates the heat, pixel by pixel (see Figure A2). As seen from the example, in these experimental conditions, even using a window of 20 pixels still results in a distribution which looks the same as when using lower values. Thus, the choice of this particular parameter may depend on the user’s preferences. In the exemplified analysis, the value of 5 pixels was chosen, since it reduces significantly the duration of the analysis, while preserving a satisfactory resolution of the heat map.

### Appendix A.4. Sampling Factor

This parameter restricts the frames chosen for analysis, and affects the heat map appearance. The example video was processed with different sampling factors, i.e., 1; 5; 10 and 25 frames, and the outcomes are presented in supplementary files with their corresponding number in brackets (Videos S13–S16). Similar to the window size parameter, this one depends on the specific user’s preferences. In particular, the heat map video will look smoother if a higher number of frames are interpolated, although some temporal changes may be masked if the cells move too fast. In the represented examples, a sampling factor of 10 frames was considered as the most appropriate.

### Appendix A.5. Maximal Accumulation

The module allows the user to interactively select and calculate the accumulation effect in different regions of a video. The module currently has two options in order to visualize the cell distribution through each frame. This affects the video appearance and the criteria used by a user to compare different heat maps resulting from different analyses. In the first option, each frame is analyzed separately, which biases the color code relationships over time, i.e., the same color in two different frames does not mean the same accumulation effect. The other option is to set a maximum value that is constant over the time of a particular video. With this approach, the color code will correspond to the same accumulation effect. This constant maximum value can be set automatically or manually, and the latter is useful for comparing multiple videos. Supplementary videos show examples of choosing constant (Videos S17 and S18) and dynamic maximum accumulation (Videos S19 and S20). Changes in the heat map in the example Video S18 demonstrate overall “heat” increasing with time. This could be explained by the fact that more spermatozoa are coming to the focus plane after the motility activation over time that in turn increases the total number of cells over the whole observation field. This behavior cannot be visualized if dynamic accumulation is used, since the maximum is calculated for each separate frame interval. Unlike the videos which represent the accumulation of the cells in a specific area close to a needle (Videos S17 and S19), in the control condition we do not see any accumulation (Videos S18 and S20). Here the slowly moving spermatozoa and no-motile cells are displaced by a flow away from the needle, resulting in the appearance of a specific “ridge” or “halo” with a low concentration of cells inside.
